# Layered Antibiofouling Composite Membrane for Quenching Bacterial Signaling

**DOI:** 10.3390/membranes12030296

**Published:** 2022-03-04

**Authors:** Kibaek Lee, Syed Salman Ali Shah, Hyeona Park, Chung-Hak Lee, Kwang-Ho Choo

**Affiliations:** 1 Department of Biotechnology and Bioengineering, Chonnam National University, Gwangju 61186, Korea; kibaek@jnu.ac.kr; 2 School of Architectural, Civil, Environmental and Energy Engineering, Kyungpook National University, Daegu 41566, Korea; salmankakakhel@yahoo.com (S.S.A.S.); gusdk07727@naver.com (H.P.); 3 Advanced Institute of Water Industry, Kyungpook National University, Daegu 41566, Korea; 4 School of Chemical and Biological Engineering, Seoul National University, Seoul 08826, Korea; leech@snu.ac.kr; 5 Department of Environmental Engineering, Kyungpook National University, Daegu 41566, Korea

**Keywords:** biofouling, layered membrane, membrane bioreactor, QQ membrane, quorum quenching, quorum sensing

## Abstract

Bacterial quorum quenching (QQ) media with various structures (e.g., bead, cylinder, hollow cylinder, and sheet), which impart biofouling mitigation in membrane bioreactors (MBRs), have been reported. However, there has been a continuous demand for membranes with QQ capability. Thus, herein, we report a novel double-layered membrane comprising an outer layer containing a QQ bacterium (BH4 strain) on the polysulfone hollow fiber membrane. The double-layered composite membrane significantly inhibits biofilm formation (i.e., the biofilm density decreases by ~58%), biopolymer accumulation (e.g., polysaccharide), and signal molecule concentration (which decreases by ~38%) on the membrane surface. The transmembrane pressure buildup to 50 kPa of the BH4-embedded membrane (17.8 h ± 1.1) is delayed by more than thrice (*p* < 0.05) of the control with no BH4 in the membrane’s outer layer (5.5 h ± 0.8). This finding provides new insight into fabricating antibiofouling membranes with a self-regulating property against biofilm growth.

## 1. Introduction

Over the past 30 years, membrane bioreactor (MBR) markets have rapidly grown with tremendous increases in the number and capacity of operating MBRs worldwide [1]. However, the biofouling caused by microbial adhesion and growth on the membrane surface limits the proliferation of MBRs, which must be overcome [2]. Several approaches have been performed to minimize the biofouling phenomena during MBR operations. Some of the most popular techniques used so far are intensive aeration and periodic maintenance cleaning (i.e., chemically enhanced cleaning) [3,4]. However, a large amount of air supply accompanies the significant energy demand. In fact, approximately 50% of the total energy required for MBRs is consumed by membrane aeration [5]. Although maintenance cleaning with active chlorine helped to delay membrane fouling, biofouling seemed to inevitably occur in MBRs [4], possibly due to the biofilm formation of chlorine-resistant bacterial species [6,7]. Neither physical nor chemical methodologies have been found to be sufficient in controlling MBR biofouling. It was thus assumed that biological methods, which can regulate microbial interactions, may open a new horizon in biofouling mitigation as reported in microbial signaling pathways [8].

Yeon, et al. [9] novelly reported that quorum sensing (QS) majorly causes membrane biofouling and that its inhibition with quorum quenching (QQ) could effectively address the biofouling issue [10]. Since then, various QQ strategies have been developed and applied in MBRs to alleviate membrane biofouling [8,11,12]. Several QQ media (e.g., spherical bead, porous silica, cylinder, hollow cylinder, and sheet), in which QQ enzymes, bacteria, or fungi are immobilized and degrade the QS signal molecules [13,14,15,16,17,18,19,20], were fabricated and applied in MBRs. These approaches can protect them from external environmental factors, including predators and competitors present in the activated sludge, thereby maintaining the QQ effect in the reactor.

**Figure 1:**
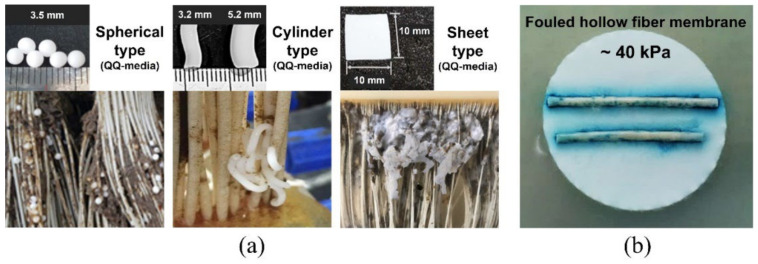
Examples of (**a**) various types (bead, cylinder, and sheet) of quorum quenching (QQ) media trapped between HFMs reprinted from references [15,21] by permission of the publisher (Taylor & Francis Ltd.). (**b**) Detection of the bacterial quorum sensing (QS) signal AHLs present at the surface of the fouled HFM [9].

However, applying the QQ media to full-scale MBRs has some challenges, as follows: (I) they can be trapped between hollow fiber membranes (HFMs) or accumulated at the dead zone of the membrane reactor (Figure 1a) [21], (II) periodical membrane cleaning is thus required to recover or remove them, and (III) an additional separation device is needed to retain the QQ media inside the MBR tank. Moreover, bacterial QS signal molecules (e.g., acyl homoserine lactones (AHLs)) remain on the membrane surface during long-term MBR operations despite applying QQ media (Figure 1b). Recently, a novel attempt to immobilize QQ bacteria on a polymeric membrane was made by using hydrophilic polymers, which reduced membrane biofouling [22]. In this study, the attachment of QQ bacteria was made by placing polymeric membranes into a mixture of QQ strains (e.g., BH4) and hydrophilic polymers for a certain period of time (6 h). The coverage and uniformity of QQ bacteria immobilized across the membrane seemed to be limited with the approach. Thus, new technologies need to be further developed, such as how to stably and simultaneously embed live QQ bacteria during the process of fabricating a polymeric membrane.

Therefore, the focus of this proof-of-concept study was to fabricate and test a novel double-layered QQ membrane comprising an outer layer containing a QQ bacterium (BH4 strain) on top of the polysulfone HFM. The layered membrane structure was examined to locate live BH4 cells and check their QQ activities through bioassays. Moreover, the growth of the biofilm-forming bacteria (e.g., *P. aeruginosa* PAO1) and accumulation of biopolymers (e.g., polysaccharides) on the membrane surface were evaluated in the presence and absence of BH4 strains in the outer layer of the composite membrane. Finally, the two different layered composite membranes with and without BH4 in the outer layer were tested in MBRs to compare their biofouling behavior.

## 2. Materials and Methods

### 2.1. Chemicals

Polysulfone (MW 7500 Da, Udel^®^ P-3500 LCD) was obtained from Solvay, Brussels, Belgium. Polyvinylpyrrolidone (PVP, MW 58,000 Da) and *N*,*N*-dimethylacetamide (DMAc, 99.8%) were purchased from Alfa Aesar, Ward Hill, MA, USA. Polyvinyl alcohol (PVA, polymerization degree = 2000) and sodium alginate were purchased from Wako, Osaka, Japan and Junsei, Tokyo, Japan, respectively. Luria-Bertani (LB) media (broth and agar) were obtained from Difco, Franklin Lakes, NJ, USA. *N*-Octanoyl-L-homoserine lactone (C8-HSL), *N*-(3-oxododecanoyl)-L-homoserine lactone (3-oxo-C12-HSL), Tris-HCl buffer (1 M, pH 7.00), and two antibiotics (tetracycline and spectinomycin) were purchased from Sigma-Aldrich, St. Louis, MO, USA. Calcium chloride, boric acid, and sodium sulfate were obtained from Daejung, Siheung-si, Korea. To detect the beta-galactosidase activity, 5-bromo-4-chloro-3-indolyl β-D-galactopyranoside (X-gal, GoldBio, St Louis, MO, USA) was used as the substrate. The beta-glo reagent for luminescence cell bioassay was purchased from Promega, Madison, WI, USA. Syto 9 (Molecular Probes, Eugene, OR, USA) and Concanavalin A (Con A, Invitrogen, Waltham, MA, USA) fluorescent dyes were used for the respective staining of BH4 cells and polysaccharides. All other chemicals used were of analytical grade.

### 2.2. Bacterial Strains

*Rhodococcus* sp. BH4 was used as a QQ bacterium because it has been reported to effectively control biofouling in MBRs [3]. *Agrobacterium tumefaciens* A136 (Ti^−^)(pCF218)(pCF372) was used as an AHL reporter strain. The strain A136 contains two plasmids, pCF218 and pCF372, that encode an AHL-responsive transcription factor traR and a traR-regulated traI-lacZ reporter, respectively. It confers resistance to 4.5 and 50 µg tetracycline/mL and spectinomycin/mL culture media, respectively. *Pseudomonas aeruginosa* PAO1-GFP, a multicopy vector encoding the green fluorescent protein (GFP), which has the carbenicillin resistance (200 µg carbenicillin/mL culture media) gene encoded on a plasmid, was used as a model strain for the AHL QS bacteria [23].

### 2.3. Preparation of Layered Composite Membranes for Bacterial QS Inhibition

**Figure 2:**
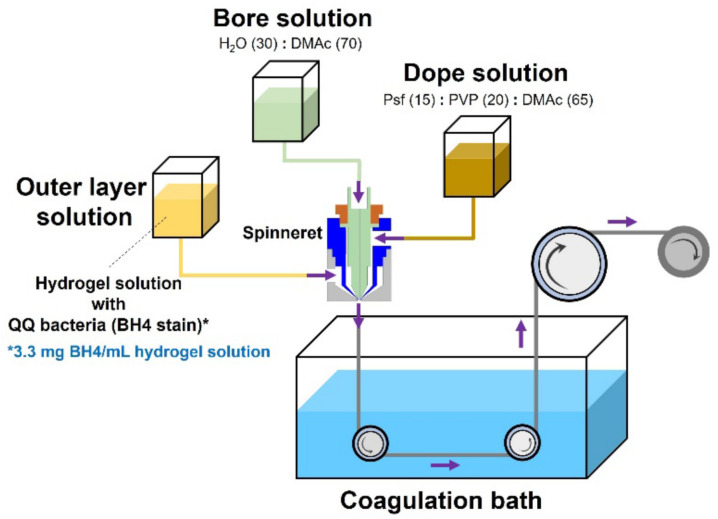
Schematic of fabrication of the double-layered membrane comprising an outer layer containing QQ bacteria (BH4 strain) and an inner polysulfone (Psf) hollow fiber.

For fabricating the double-layered membranes (inner layer is polysulfone, and outer layer is hydrogel), a dope solution was prepared by dissolving polysulfone (15 wt/wt%) and PVP (a hydrophilic additive, 20 wt/wt%) in dimethylacetamide (DMAc, 65 wt/wt%) with shaking (50 rpm) at 60 °C overnight. Moreover, a bore solution comprising a 7:3 mass ratio of DMAc and H_2_O was prepared [24,25]. The hydrogel solution containing PVA (10 wt/wt%) and sodium alginate (1 wt/wt%) was prepared and sterilized in an autoclave for 15 min at 121 °C. More specifically, a bore solution (70 wt/wt% DMAc) was discharged through the inside of a 0.5 mm–diameter spinneret at an extrusion rate of 1 mL/min, using a syringe pump. The dope solution was pumped through the middle side of a 2.0 mm inner–diameter spinneret at the same flow rate (1 mL/min). The hydrogel solution (PVA and sodium alginate) was ejected over the dope solution through the outermost spinneret with a 2.8 mm inner diameter. The ejected double-layered composite membrane was soaked in the coagulation bath (7 wt/wt% boric acid and 4 wt/wt% CaCl_2_) for 2 h (Figure 2). Afterward, it was transferred to a sodium sulfate solution (0.5 M) and soaked for an additional 8 h. BH4 (3.3 mg/mL) was added to the hydrogel solution for an outer layer containing BH4 cells (designated as the BH4-embedded membrane). An equivalent amount of pure water was added instead to prepare a BH4-free membrane (the control). To produce the HFM module that can be tested in an MBR, the lab-made membranes were immersed in glycerol (25 wt/wt%) for 12 h. After drying them, a bundle of the hollow fibers was put together and glued with epoxy resin.

### 2.4. Assessment of QQ Activity of Layered Composite Membranes

#### 2.4.1. Bioassay by Cross Feeding Method

The QQ activity defined by the inhibition of bacterial QS signaling by the layered composite membranes was determined from the degradation of C8-HSL, a dominant AHL QS signal molecule in the activated sludge of the MBRs [9]. C8-HSL was assessed through a cell bioassay of the beta-galactosidase activity of the AHL reporter strain, *A. tumefaciens* A136, which produces beta-galactosidase depending on the concentration of AHLs [15]. Briefly, the strain A136 was inoculated in a half-arrow shape on both edges of the LB agar plate pre-spread with X-gal (20 μg/mL). C8-HSL (200 nM) was placed in the center, and each piece of the BH4-free and BH4-embedded layered membranes was placed between C8-HSL and A136, respectively. The QQ activity was determined according to whether the AHL (C8-HSL) placed in the center can diffuse to the reporter strain A136 passing through each membrane. It can be assumed that if C8-HSL is undegraded by the membrane, it will reach A136, eventually producing a blue color.

#### 2.4.2. Bioassay by Luminescence Method

The cultured strain A136 was diluted in freshly sterilized LB broth with two antibiotics (4.5 μg/mL tetracycline and 50 μg/mL spectinomycin) to obtain an optical density of 0.1 at a 600 nm wavelength (OD_600_). Then 95 μL diluted strain A136 and 5 μL of each sample or standard AHL (i.e., 3-oxo-C12-HSL) were loaded into each well of a 96-well plate, which was covered by a plastic film to prevent evaporation and then incubated at 30 °C for 90 min without shaking. Subsequently, 30 μL per well of beta-glo reagent were supplemented, and the plate was stored at 25 °C for 40 min with a foil cover to prevent photobleaching. The bioluminescent intensity of each well was measured by using a luminometer (Synergy 2, BioTek, Winooski, VT, USA), and the AHL concentration was determined from a calibration curve formed by using 3-oxo-C12-HSL as a standard.

### 2.5. MBR Operating Conditions

Lab-scale MBRs were operated and fed with typical synthetic wastewater, as described in a previous study [22]. The HFM module prepared as described above was installed in the MBRs. The transmembrane pressure (TMP) was monitored and recorded until it reached 50 kPa when the MBR systems were stopped. The extent of membrane biofouling was evaluated based on the TMP buildup over time. The reactor configuration and operating conditions are detailed in later sections.

### 2.6. Analytical Methods

Confocal laser canning microscope (CLSM) and scanning electron microscope (SEM) analyses of layered composite membranes were performed by using CLSM (LSM 700, Carl Zeiss, Jena, Germany) and SEM (SU-8220, Hitachi, Tokyo, Japan), respectively. The membrane specimens for CLSM and SEM analyses were prepared as described in a previous study [22].

### 2.7. Statistical Analysis

All analytical experiments were conducted at least in triplicates (*n* = 3). Error bars represent one standard deviation. Furthermore, the *p*-values were obtained from the two-tailed Student’s *t*-test.

## 3. Results and Discussion

### 3.1. Visualization of Composite Membranes Comprising BH4-Free or BH4-Embedded Outer Layers

**Figure 3:**
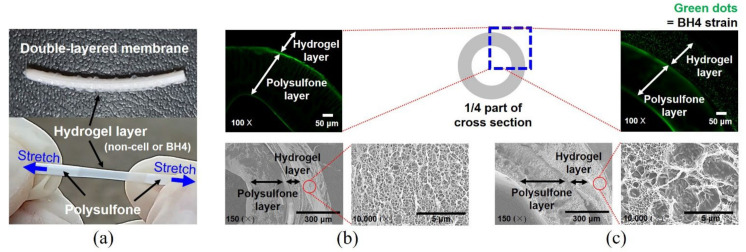
Double-layered composite membranes. (**a**) A photo of the double-layered membrane prepared by the experimental setup given in Figure 2. The composite membrane comprising outer (i.e., hydrogel) and inner (i.e., polysulfone) layers. Visualization (CLSM and SEM) of (**b**) BH4-free layered membrane and (**c**) BH4-embedded layered membrane. The fluorescent dye (Syto 9) was used for staining the BH4 cells, which are shown in green dots in (**c**). The membrane edge looks green due to dye adsorption.

Figure 3a shows a double-layered membrane comprising (I) the outer hydrogel layer, where QQ bacteria (i.e., BH4 strain) are embedded; and (II) the inner polysulfone membrane layer surrounded by the hydrogel layer. The outer hydrogel layer was a bit bumpy and relatively elastic compared to the inner polysulfone hollow fiber. To visualize the live BH4 cells embedded inside the hydrogel layer above the polysulfone fiber, the fluorescent dye (Syto 9) was applied. Thus, the live BH4 cells are green-colored. The cross-section of the BH4-free layered membrane (the control) is shown in black (except the green light by dye adsorption at the membrane edge), and empty pores exist in the hydrogel layer as observed by the CLSM and SEM images, respectively (Figure 3b). However, the live BH4 cells embedded inside the hydrogel layer were seen as green dots by the CLSM analysis. In addition, their presence was also observed in the hydrogel matrix by using SEM analysis (Figure 3c). The result demonstrates that live BH4 strains were successfully immobilized into the outer layer of the hydrogel–polysulfone composite membrane.

### 3.2. Quorum Quenching Activity and Antibiofouling Effect of Layered QQ Composite Membrane

**Figure 4:**
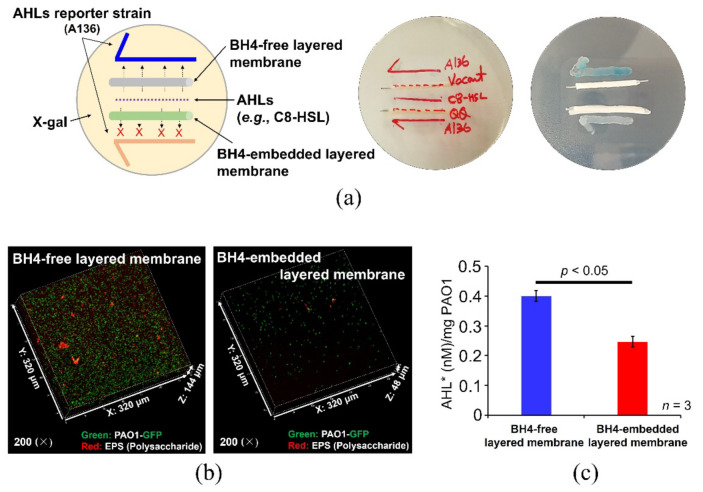
QQ activity and antibiofouling effects of the layered composite membranes. (**a**) AHL (e.g., C8-HSL) quenching by BH4 in the layered membrane. (**b**) CLSM images of BH4-free and BH4-containing layered membrane surfaces. The fluorescent dye, Concanavalin A, was used for staining. Polysaccharides are shown in red. The green color indicates the growth of GFP-tagged *P. aeruginosa* PAO1, a model strain for AHL QS bacterium. (**c**) Concentration of AHL* (3-oxo-C12-HSL) was detected from the surface of the two layered membranes (*n* = 3). The error bars represent one standard deviation, and the *p*-value is derived from a two-tailed Student’s *t*-test.

To evaluate the degradation (i.e., QQ activity) of the dominant AHL, C8-HSL, by the BH4-embedded membrane, bioassay tests were conducted. C8-HSL was placed in the center of the agar medium with X-gal, and its color change was checked by the AHL reporter strain (strain A136, inoculated in half arrow shape) when BH4-free and BH4-embedded membranes were placed between them (Figure 4a). For the BH4-free membrane, C8-HSL freely diffused and reached the strain A136 (as shown in blue), whereas, for the BH4-embedded layered membrane, C8-HSL appeared to be decomposed by the BH4 cells present in the hydrogel layer and, thereby, did not reach A136 (as indicated by no change in color).

To assess the antibiofouling effect of the BH4-embedded layered membrane, the degree of biofilm formation and the AHL concentration (i.e., 3-oxo-C12-HSL) produced were quantified by using *P. aeruginosa* PAO1 tagged GFP as a model AHL QS bacterium. In brief, each membrane prepared in Section 2.3 was incubated in LB broth inoculated with strain PAO1-GFP at 37 °C for 48 h with orbital shaking (200 rpm). Then, the membrane was taken out, stained with Con A, red fluorescent dye, for polysaccharides, and observed with CLSM. The CLSM analysis results (Figure 4b) show that the degree of biofilm formation was significantly reduced on the BH4-embedded membrane surface. The density (*z*-axis) of PAO1-GFP (shown in green), which accumulated on the membrane surface, decreased from 114 to 48 μm (i.e., by ~58%), and the amount of polysaccharides (shown in red), which is considered an essential component of EPSs, was also remarkably reduced by the BH4-embedded membrane. Moreover, it was shown that the production of AHL (3-oxo-C12-HSL) per unit PAO1 cell mass decreased by ~38% with the BH4-embedded layered membrane (0.25 nM ± 0.018/mg PAO1) than the BH4-free layered membrane (0.40 nM ± 0.019/mg PAO1) (Figure 4c). The result indicates that (I) the QS signaling among PAO1 strains was inhibited by BH4 strains embedded in the outer layer; and (II) polysaccharides, which act as molecular glue in biofilm formation, was less secreted in the presence of BH4 strains. Thus, it is believed that the BH4 strains embedded in the outer layer can successfully control bacterial QS, potentially rendering an antibiofouling effect.

### 3.3. Performance of BH4-Embedded Layered Membrane in MBR

**Figure 5:**
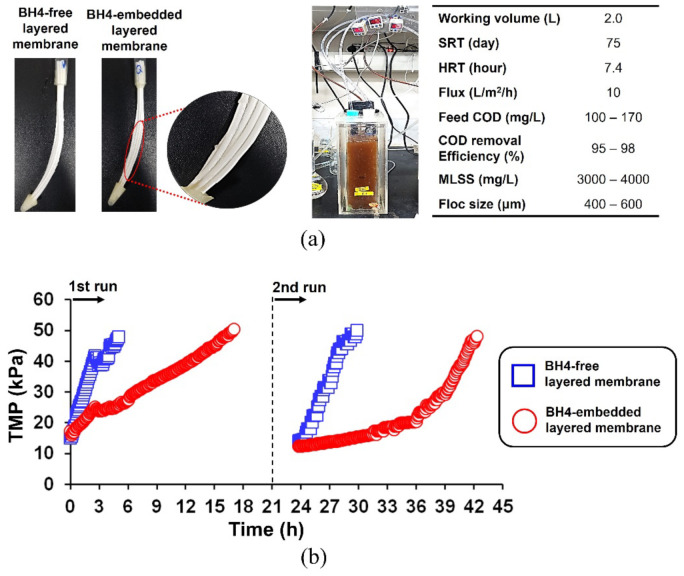
Application of BH4-free and BH4-embedded layered membranes in MBRs. (**a**) Images of BH4-free and BH4-embedded layered membrane modules used for laboratory MBRs, which were operated under the given conditions. (**b**) TMP profiles of two types of layered composite membranes. New membrane modules were employed for each run.

To evaluate the effect of the BH4 strains embedded in the layered membrane on biofouling control, the MBR fed with synthetic wastewater was operated under the conditions (Figure 5a). The rate of TMP buildup, which reflects the progress of membrane biofouling, was significantly mitigated when BH4 was embedded in the outer layer of the composite membrane. During the first run, the time required for the TMP to reach 50 kPa was 5.0 and 17.0 h for the BH4-free and BH4-embedded membranes, respectively, indicating that BH4 in the layered membrane substantially delayed membrane biofouling by more than thrice that of the control (which has no BH4 in the outer layer).

To check the reproducibility of the above result, another run was conducted under the same operating conditions as the first run. The TMP buildup of the BH4-free membrane rapidly increased to 50 kPa within 6.1 h, whereas that of the BH4-embedded membrane (18.6 h) increased slowly (*p* < 0.05, Figure 5b). The result demonstrates that the QQ bacteria (BH4 strain) in the outer layer of the composite membrane mitigated biofouling as effectively as or exceeding those in the QQ media (e.g., microbial vessel, spherical bead, silica, cylinder, hollow cylinder, and sheet), which alleviated membrane biofouling as reported in previous QQ studies [8,11]. Notwithstanding this trend, the relatively high initial TMP level (i.e., low water permeability), caused by the formation of the outer hydrogel layer containing BH4 strains, must be addressed in future studies. The stability of the BH4-embedded hydrogel layer also needs to be explored by employing long-term tests with various membrane cleanings regarding the practicality of the BH4-embedded layered membrane.

## 4. Conclusions

Herein, AHLs (e.g., C8-HSL and 3-oxo-C12-HSL) were effectively degraded by BH4-embedded composite membrane. The cell adhesion (~58%), EPS (e.g., polysaccharides) production, and AHL concentration (~38%) were significantly reduced in the BH4-embedded membrane when *P. aeruginosa* PAO1-GFP was used as an AHL QS bacterium. The membrane biofouling time significantly increased from 5.5 ± 0.8 h to 17.8 ± 1.1 h with the BH4-embedded membrane (i.e., biofouling was mitigated by a factor of >3; *p* < 0.05) when the membrane was applied to the MBR fed with synthetic wastewater. However, some challenges remain to be resolved in future studies, such as (I) lowering the initial high TMP caused by the formation of the outer layer comprising BH4 strains embedded in the hydrogel layer on top of the polysulfone membrane and (II) maintaining the QQ activity and stability of the BH4-embedded layer during long-term use with membrane cleaning. Nevertheless, this novel approach elucidates how effectively QQ bacteria (BH4) embedded in the composite membrane reduced membrane biofouling. Thus, further developments of QQ-based antibiofouling membranes are expected.

## Data Availability

The data that support the findings of this study are available from the corresponding author.

## References

[1] Al-Asheh S., Bagheri M., Aidan A. (2021). Membrane bioreactor for wastewater treatment: A review. Case Stud. Chem. Environ. Eng..

[2] Aslam M., Ahmad R., Kim J. (2018). Recent developments in biofouling control in membrane bioreactors for domestic wastewater treatment. Sep. Purif. Technol..

[3] Weerasekara N.A., Choo K.-H., Lee C.-H. (2014). Hybridization of physical cleaning and quorum quenching to minimize membrane biofouling and energy consumption in a membrane bioreactor. Water Res..

[4] Weerasekara N.A., Choo K.-H., Lee C.-H. (2016). Biofouling control: Bacterial quorum quenching versus chlorination in membrane bioreactors. Water Res..

[5] Krzeminski P., van der Graaf J.H., van Lier J.B. (2012). Specific energy consumption of membrane bioreactor (MBR) for sewage treatment. Water Sci. Technol..

[6] Bhojani G., Binod Kumar S., Kumar Saha N., Haldar S. (2018). Membrane biofouling by chlorine resistant Bacillus spp.: Effect of feedwater chlorination on bacteria and membrane biofouling. Biofouling.

[7] Song Y., Mao G., Gao G., Bartlam M., Wang Y. (2019). Resistance and resilience of representative low nucleic acid-content bacteria to free chlorine exposure. J. Hazard. Mater..

[8] Lee K., Yu H., Zhang X., Choo K.-H. (2018). Quorum sensing and quenching in membrane bioreactors: Opportunities and challenges for biofouling control. Bioresour. Technol..

[9] Yeon K.-M., Cheong W.-S., Oh H.-S., Lee W.-N., Hwang B.-K., Lee C.-H., Beyenal H., Lewandowski Z. (2008). Quorum sensing: A new biofouling control paradigm in a membrane bioreactor for advanced wastewater treatment. Environ. Sci. Technol..

[10] Yeon K.-M., Lee C.-H., Kim J. (2009). Magnetic enzyme carrier for effective biofouling control in the membrane bioreactor based on enzymatic quorum quenching. Environ. Sci. Technol..

[11] Oh H.-S., Lee C.-H. (2018). Origin and evolution of quorum quenching technology for biofouling control in MBRs for wastewater treatment. J. Membr. Sci..

[12] Köse-Mutlu B., Ergön-Can T., Koyuncu I., Lee C.-H. (2019). Quorum quenching for effective control of biofouling in membrane bioreactor: A comprehensive review of approaches, applications, and challenges. Environ. Eng. Res..

[13] Oh H.-S., Yeon K.-M., Yang C.-S., Kim S.-R., Lee C.-H., Park S.Y., Han J.Y., Lee J.-K. (2012). Control of membrane biofouling in MBR for wastewater treatment by quorum quenching bacteria encapsulated in microporous membrane. Environ. Sci. Technol..

[14] Kim S.-R., Oh H.-S., Jo S.-J., Yeon K.-M., Lee C.-H., Lim D.-J., Lee C.-H., Lee J.-K. (2013). Biofouling control with bead-entrapped quorum quenching bacteria in membrane bioreactors: Physical and biological effects. Environ. Sci. Technol..

[15] Lee K., Choo K.-H., Ng H.Y., Lee C.-H. (2020). Preparation of a mesoporous silica quorum quenching medium for wastewater treatment using a membrane bioreactor. Biofouling.

[16] Lee S., Lee S.H., Lee K., Kwon H., Nahm C.H., Lee C.-H., Park P.-K., Choo K.-H., Lee J.-K., Oh H.-S. (2016). Effect of the shape and size of quorum-quenching media on biofouling control in membrane bioreactors for wastewater treatment. J. Microbiol. Biotechnol..

[17] Lee S.H., Lee S., Lee K., Nahm C.H., Kwon H., Oh H.-S., Won Y.-J., Choo K.-H., Lee C.-H., Park P.-K. (2016). More efficient media design for enhanced biofouling control in a membrane bioreactor: Quorum quenching bacteria entrapping hollow cylinder. Environ. Sci. Technol..

[18] Nahm C.H., Choi D.-C., Kwon H., Lee S., Lee S.H., Lee K., Choo K.-H., Lee J.-K., Lee C.-H., Park P.-K. (2017). Application of quorum quenching bacteria entrapping sheets to enhance biofouling control in a membrane bioreactor with a hollow fiber module. J. Membr. Sci..

[19] Iqbal T., Shah S.S.A., Lee K., Choo K.-H. (2021). Porous shell quorum quenching balls for enhanced anti-biofouling efficacy and media durability in membrane bioreactors. Chem. Eng. J..

[20] Lee K., Lee S., Lee S.H., Kim S.-R., Oh H.-S., Park P.-K., Choo K.-H., Kim Y.-W., Lee J.-K., Lee C.-H. (2016). Fungal quorum quenching: A paradigm shift for energy savings in membrane bioreactor (MBR) for wastewater treatment. Environ. Sci. Technol..

[21] Lee K., Park J.-S., Iqbal T., Nahm C.H., Park P.-K., Choo K.-H. (2018). Membrane biofouling behaviors at cold temperatures in pilot-scale hollow fiber membrane bioreactors with quorum quenching. Biofouling.

[22] Shah S.S.A., Lee K., Park H., Choo K.-H. (2022). Live membrane filters with immobilized quorum quenching bacterial strains for anti-biofouling. J. Membr. Sci..

[23] Kim J.-H., Choi D.-C., Yeon K.-M., Kim S.-R., Lee C.-H. (2011). Enzyme-immobilized nanofiltration membrane to mitigate biofouling based on quorum quenching. Environ. Sci. Technol..

[24] Ohya H., Shiki S., Kawakami H. (2009). Fabrication study of polysulfone hollow-fiber microfiltration membranes: Optimal dope viscosity for nucleation and growth. J. Membr. Sci..

[25] Choi S.H., Chung J.W., Priestley R.D., Kwak S.-Y. (2012). Functionalization of polysulfone hollow fiber membranes with amphiphilic β-cyclodextrin and their applications for the removal of endocrine disrupting plasticizer. J. Membr. Sci..

